# MAGERI: Computational pipeline for molecular-barcoded targeted resequencing

**DOI:** 10.1371/journal.pcbi.1005480

**Published:** 2017-05-05

**Authors:** Mikhail Shugay, Andrew R. Zaretsky, Dmitriy A. Shagin, Irina A. Shagina, Ivan A. Volchenkov, Andrew A. Shelenkov, Mikhail Y. Lebedin, Dmitriy V. Bagaev, Sergey Lukyanov, Dmitriy M. Chudakov

**Affiliations:** 1 Shemyakin-Ovchinnikov Institute of bioorganic chemistry RAS, Miklukho-Maklaya 16/10, Moscow, Russia; 2 Pirogov Russian National Research Medical University, Ostrovityanova 1, Moscow, Russia; 3 Central European Institute of Technology, Masaryk University, Brno, Czech republic; 4 Evrogen JSC, Miklukho-Maklaya 16/10, Moscow, Russia; 5 Skolkovo Institute of Science and Technology, Nobel 3, Moscow, Russia; University of Canterbury, NEW ZEALAND

## Abstract

Unique molecular identifiers (UMIs) show outstanding performance in targeted high-throughput resequencing, being the most promising approach for the accurate identification of rare variants in complex DNA samples. This approach has application in multiple areas, including cancer diagnostics, thus demanding dedicated software and algorithms. Here we introduce MAGERI, a computational pipeline that efficiently handles all caveats of UMI-based analysis to obtain high-fidelity mutation profiles and call ultra-rare variants. Using an extensive set of benchmark datasets including gold-standard biological samples with known variant frequencies, cell-free DNA from tumor patient blood samples and publicly available UMI-encoded datasets we demonstrate that our method is both robust and efficient in calling rare variants. The versatility of our software is supported by accurate results obtained for both tumor DNA and viral RNA samples in datasets prepared using three different UMI-based protocols.

This is a *PLOS Computational Biology* Software paper.

## Introduction

The ability to infer rare variants is important for a large domain of high-throughput genome re-sequencing applications: cancer [1] and prenatal [2] diagnostics, studies of tumor heterogeneity and variability [3], bacterial [4] and viral [5] drug resistance, as well as microbiome profiling[6] and basic evolutionary studies [7]. The detection of rare variants is also crucial for clinical applications such as early detection of cancer and monitoring of its progression [8,9].

Conventional pipelines, however, do not suit well for the detection of ultra-rare mutations. Current tools were shown to reliably detect mutations present at ~5% in real data[10–12], while practical applications such as cancer detection require searching for rare mutations present at a rate of ~0.1% [8,13–17]. As additional complication, commonly used variant calling tools do not perform well in ultra-high (> 1,000x) coverage setting [10], which is a prerequisite to achieve the desired accuracy for mutations with less than 1% frequency. Rare variant detection capability is also limited by sequencing errors and sampling/library preparation biases [18], requiring custom molecular assays [17,19] to reach the desired accuracy level.

Recently introduced unique molecular identifier (UMI) tagging approach[20–22] shows outstanding performance in targeted re-sequencing experiments and facilitates (c)DNA molecule quantification, and elimination of PCR and sequencing errors. While UMI tagging approach was extensively used in a large number of recent studies [6,16,21–30], there is still no dedicated software pipeline able to efficiently process UMI-tagged targeted re-sequencing data.

At the same time, adapting existing software to the analysis of UMI-tagged data is unfeasible. For example, conventional software tools heavily rely on sequencing quality values to estimate error rates at variant calling stage. Error frequencies, however, are not that straightforward to infer for UMI-assembled consensuses. Moreover, even after consensus assembly, the data is rich for seemingly high-quality errors that are inevitable when using PCR to perform UMI tagging and can arise from 1st cycle PCR errors [21]. This problem is of high importance and must be solved in order to implement a variant calling algorithm suitable for UMI-tagged data.

**Fig 1:**
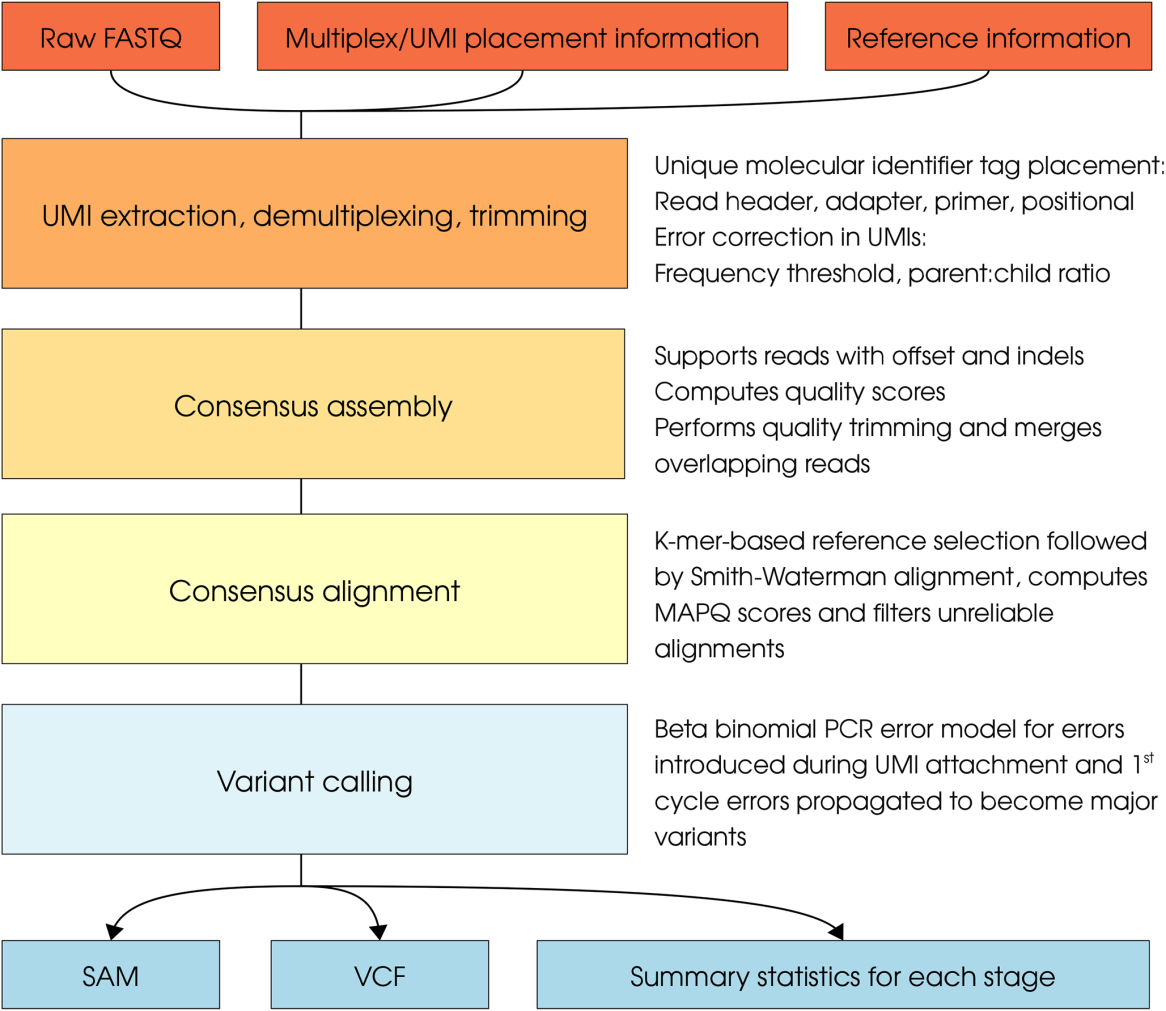
MAGERI pipeline. The figure describes four steps implemented in MAGERI pipeline. The pipeline starts with raw FASTQ files (either single- or paired-end), UMI tagging information (such as primer and adapter sequences containing random N bases, or the coordinates of N bases in raw reads) and reference information (FASTA file, BED file with genomic coordinates and contig information. UMIs are extracted from raw reads and used to group reads into molecular identifier groups (MIGs) which are then assembled into consensus sequences. Consensus sequences are then mapped to corresponding references, variant calling is performed and MAGERI Q scores are computed for substitutions using a Beta-Binomial model that accounts for PCR errors introduced during UMI tagging step in case UMIs are attached using PCR or RT-PCR, or 1st cycle PCR errors in case UMIs are attached using ligation.

Here we introduce MAGERI (Molecular tAgged GEnome Re-sequencing pIpeline), a dedicated software tool that implements UMI tag extraction and processing routines, an assembly routine that groups sequencing reads tagged with the same UMI into consensuses, and consensus alignment and variant calling modules (**Fig 1**). The pipeline corrects errors in the UMI sequences and performs fast and robust consensus assembly able to handle reads with high error load, indels and random offsets. It also takes an advantage of data reduction by consensus assembly and a priori knowledge of target region positions [31] to run a highly sensitive alignment algorithm. As UMI correction removes nearly all sequencing errors, MAGERI implements a variant quality scoring model that accounts for PCR errors introduced at the UMI attachment stage and 1st cycle PCR errors that can propagate to become dominant variants in the consensus sequence. A comprehensive benchmark of MAGERI software is performed using a diverse set of high-throughput sequencing datasets that employ UMI-tagging approach listed in **Table 1**.

**Table 1:** Datasets used for MAGERI benchmark.

Dataset	Source	UMI tagging method	Sequencing method	Control variants
Tru-Q 7	This study	Linear PCR	Illumina HiSeq	27 substitutions, 1 deletion. Variant frequency 1–30%, see **S2 Table** for details
Tru-Q 7, 1:9 diluted with healthy donor PBMC DNA	This study	Linear PCR	Illumina HiSeq	27 substitutions, 1 deletion. Variant frequency 0.1–3%, see **S2 Table** for details
Healthy donor PBMC DNA	This study	Linear PCR	Illumina HiSeq	None, all variants are either allelic or erroneous
Tumor and plasma DNA from two cancer patients	This study	Linear PCR	Illumina HiSeq	BRAF V600E in tumor
Duplex sequencing	[43]	Ligation	Illumina HiSeq	ABL1 E279K at 1% frequency
HIV amplicon sequencing	[37]	RT-PCR	Illumina MiSeq	N/A*
Torrent	[38]	PCR	Ion Torrent	N/A**

*—no intrinsic control variants available. Two samples were used: supernatant from 8E5 cell line that should yield unmutated HIV cDNA and HIV cDNA from patient plasma.

**—only sequencing data for a single template with no appropriate variants is publicly available

## Materials and methods

### Ethics statement

Tumor and blood samples from patients with malignant melanoma were collected at Molecular Biology & Cytogenetics Lab, Russian Center for Roentgenology & Radiology (Moscow, Russian Federation). The study was approved by the local ethics committee and conducted in accordance with the Declaration of Helsinki. All donors were informed of the final use of the samples and signed an informed consent document.

### Control DNA samples

For determination of analytical sensitivity and selectivity of the method, negative and positive control DNA samples were constructed. Negative control sample was comprised of genomic DNA extracted from PBMC of a healthy donor (kindly provided by Dr. Alexander Abramov, NPCMPD, Moscow, Russian Federation). Positive control sample was obtained by using a Tru-Q 7 1% Tier reference mutation panel (Horizon Dx, USA; Cat. ID HD734) and by mixing Tru-Q 7 1% Tier reference with negative control sample at 1: 9 ratio. Tru-Q 7 mutation panel and negative control DNA was fragmented with dsDNA Fragmentase (NEB, cat. # M0348) and dsDNA Fragmentase buffer (NEB, cat. # B0348) according to manufacturer's protocol. DNA concentration was determined by Qubit fluorometry and real-time qPCR with both methods giving comparable results. Positive control sample was constructed based on real-time qPCR data for Tru-Q 7 mutation panel and negative control. Control samples were further tested for mutations in the hotspots of *KRAS* exon 2, *NRAS* exon 3, *BRAF* exon 15 and *EGFR* exons 18–21 using Insider Mutation Detection Kits (Evrogen Lab Ltd, Moscow, Russian Federation) and TaqMan Mutation Detection Reagents (Thermo Fisher, Waltham, Massachusetts, United States). Insider Mutation Detection Kits are based on wild-type blocking PCR [32] coupled to real-time detection using two "kissing" (FRET) probes [33]. Kits’ limit of detection, specificity and selectivity as determined by manufacturer are 10 copies, 99,5% and 1% of mutant DNA.

Mutation load was found to be present in the desired range–about 0.1% per each mutation in positive control, while no mutations were detected in negative control samples. The list of Tru-Q 7 variants covered by our primer panel (listed in **S1 Table**) together with their frequencies provided by vendor is given in **S2 Table.**

### ctDNA detection samples

Paired tumor and blood samples from two patients with malignant melanoma of the skin were collected at Molecular Biology & Cytogenetics Lab, Russian Center for Roentgenology & Radiology (Moscow, Russian Federation). Blood samples were obtained 1–2 hours before surgery and processed within 40 minutes after collection. Plasma was separated from blood cells according to standard protocols as described[34] and then stored at minus 80°C. Tumor samples were provided as FFPE blocks with corresponding haematoxylin-eosin stained slides. These slides were checked for tumor presence and for consistency with the provided blocks by two certified pathologists. Afterwards, 10 6-um thick sections were cut from each block on a rotary microtome and mounted on poly-L-lysine slides. DNA was extracted from FFPE sections on slides using QiaAMP FFPE Tissue Kit (Qiagen, Hilden, Germany) according to manufacturer’s instructions with minor modifications: DNA was extracted from FFPE sections on slides using three-step procedure. First, the FFPE tissue sections were deparaffinized using 100% hexadecane (incubation at 56°C for 5 minutes) and air-dried. The slides were then moisturized with Tris-based buffer (pH 8.0) and tissue fragments were scraped off the slides using 200-ul pipette tips and put into 1.5-ml microcentrifuge tubes (Sarstedt). 500 ul of Tris-based buffer (pH 8.0) and 40 IU of Proteinase K (Amresco) were added, the tube was vortexed briefly and incubated at 56°C for 4 hours. After repeated brief vortexing QiaAMP FFPE Tissue Kit protocol was followed starting from section 14.

Circulating DNA extraction from plasma was performed on a QiaVac-24 vacuum manifold using QiaAMP Circulating Nucleic Acids Kit (Qiagen, Hilden, Germany) according to manufacturer’s protocol for 5-ml plasma samples. DNA concentration was determined by real-time qPCR. Tumor DNA samples were analyzed for mutations in the hotspots of *BRAF* exon 15 using Insider B-Raf Mutation Detection Kit (Evrogen Lab Ltd, Moscow, Russian Federation), TaqMan Mutation Detection Reagents (Thermo Fisher, Waltham, Massachusetts, United States) and both tumors were found to be *BRAF* V600E-positive.

### Libraries preparation and sequencing

UMI-tagged libraries preparation was performed as described on **S1 Fig**. To ensure robust UMI attachment, tagging of each target DNA molecule was performed using 5 cycles of linear PCR amplification, followed by two-stage exponential amplification of tagged molecules combined with attachment of Illumina sequencing adapters. Mutations in 63 “hot-spot” regions of human proto-oncogenes and tumor suppressor genes were analyzed. Region-specific primers were divided into 4 pools to ensure optimal performance of multiplexed PCR. Target region length varied from 160 to 210 bp. Full list of genes, regions, primer sequences and their distribution between the 4 pools are outlined in **S1 Table**. Efficiency of primer removal with *E*. *coli* Exonuclease I (New England Biolabs, USA) was controlled by adding a spike template (158-bp fragment of TurboFP650 fluorescent protein[35]) and primers for its amplification to each multiplex PCR pool. UMI tagging primer for this template was included in the primer mix for linear PCR amplification, whereas template itself was added only at the stage of exponential amplification. Hence successful amplification of this sequence would occur only in case of incomplete removal of UMI-tagging primers. Suppression of non-specific amplification products was achieved by concurrent use of nested and step-out PCR[36]. Sample preparation was done: for control DNA samples–in duplicate for all 4 primer pools, for tumor DNA samples–once for all 4 primer pools, for plasma DNA samples–once for primer pool 3 only (this pool includes *BRAF* exon 15 due to limited quantity of DNA). Samples were pooled and sequenced on HiSeq2500 lane using TruSeq V. 4 chemistry with 100-bp paired-end reads. List of sequenced samples and the sequencing read yield is shown in **S3 Table**.

### Software availability and implementation

MAGERI is implemented in Java v 1.8 and is distributed as a single cross-platform executable JAR file [https://github.com/mikessh/mageri]. Software documentation is available here [http://mageri.readthedocs.org/en/latest/]. Description, generated output files and scripts that can be used to reproduce the analysis performed in this paper can be found here: [https://github.com/mikessh/mageri-paper]. MAGERI is free for scientific and nonprofit use. MAGERI analysis can run on a commodity hardware in a reasonable time. For example, processing a sample of 30 million pair-end reads using a 32 GB RAM and 8-core Intel Xeon processor UNIX server takes approximately 30 minutes with the most running time consumed by I/O at the stage of primer matching and sample de-multiplexing. The analysis of duplex sequencing dataset mentioned below takes ~10 minutes using the same hardware setup. Default MAGERI parameters, scripts (R markdown templates) and MAGERI output used to perform the analysis described in this paper can be accessed at [https://github.com/mikessh/mageri-paper].

### Data pre-processing: UMI extraction

Unique molecular identifier (UMI) sequences were first extracted from raw sequencing reads, and UMIs with minimal quality (across the whole length of UMI sequence) less than a specified threshold (Phred 20) were discarded. Reads tagged with identical UMI sequence were assembled into molecular identifier groups (MIGs). On this stage, in case a pair of MIGs have a UMI sequence that differ by one or two substitutions and their relative sizes differ by 20 (400 for two substitutions)-fold the smaller MIG is considered to be tagged by an erroneous UMI sequence and discarded. Representative MIG size distribution is given at **S2A Fig**. Note that a clear size peak is seen when the distribution is weighted by read count, as small MIGs represent the majority of unique UMIs but contain a minor fraction of reads. Also note that this distribution is highly skewed, so log transformation was applied. MIGs were size-thresholded with the threshold selected to be the square root of peak position (that is, 1/2 of log-transformed peak position). Discarded MIGs represent an erroneous UMI sub-variant or PCR/sequencing artifacts. Given mismatches in the UMI sequence are corrected, one can safely use a 5 reads per UMI coverage threshold as it is enough to remove nearly all sequencing errors, unless an extremely poor sequencing quality dataset is being analyzed.

### Data pre-processing: Consensus assembly

Reads within each MIG are aligned and assembled, the major (most frequent) nucleotide at each position are combined to form the MIG consensus sequence. During the assembly procedure, “core” sequence regions (30 bases, with +/-5 base offset to read center) were extracted from each read and the most frequent core region was used to choose offset for each read. Reads that do not match the core region or have more than two consequent mismatches (likely due to indel errors) were dropped. The latter can be re-aligned using a local alignment algorithm for indel-prone 454/IonTorrent data.

Differences between individual reads and the consensus sequence summarized in order to be further used for estimation of PCR error rate. We hereafter refer to sub-variants that are present within the consensus and are different from the most frequent base at a given position as “minor” variants. We only consider bases above a certain quality threshold *Q* (e.g. Phred 30 for HiSeq or Phred 20 for longer MiSeq data that typically has lower quality) and variants having frequency above corresponding value of 10^−*Q*/10^ for the calculation.

Consensus quality score (CQS) at a given position is calculated as

$$CQS=\frac{40}{3}∙(4f-1)$$

where *f* is the frequency of a dominant nucleotide.

### Data pre-processing: Consensus sequence alignment

MIG assembly greatly reduces the effective number of sequences and allows to use a highly sensitive alignment algorithm. A two-staged alignment scheme was used: best reference sequence was selected based on K-mer matching, consensus sequence is than aligned to the best reference hit using Smith-Waterman algorithm. Local alignment parameters were set as follows: match reward of 1, mismatch penalty of -3, gap open penalty of -6 and gap extend penalty of -1. K-mer matching score is calculated as the total information content of matching K-mers,

$$I=-\sum_{k}f_{k}\;\log\;f_{k}$$

where *f*_*k*_ is the frequency of a given K-mer frequency among all K-mers in reference database.

The mapping quality score (*MAP*_*Q*_) is calculated as

$$MAP_{Q}=10∙(I_{best\;hit}-I_{next\;best\;hit})$$

to resemble *MAP*_*Q*_ scores calculated by commonly used software such as BWA and bowtie.

The performance of reference selection step was tested by simulating query sequences from homologous reference database under fixed error rates (**S4 Table**). To filter false-positive mappings we have discarded consensus sequences displaying local alignments that have less than 90% identity (accounting for substitutions only) or span less than 70% of query sequence. To benchmark our aligner on a complex case with real genomic data, we have generated reads from sequences of pseudogenes that had Cancer Gene Census (CGC) genes as parents according to pseudogene.org. We then aligned those reads to CGC gene references and observed false alignment rate of 4%. MAGERI aligner accuracy reported here is in a good agreement with aligner benchmark for targeted capture sequencing [31].

### Variant calling

Sequencing errors are the major source of false-positive variants inferred from HTS data. Conventional variant callers rely on read count distribution and sequencing quality to estimate error rate and compute variant quality scores. Rational interpretation of variant calling quality for the UMI-assembled consensuses, however, requires a different approach in order to estimate the consensus error probabilities appropriately. A straightforward way to do would be to use the frequency of major nucleotide at each given position in consensus, e.g. in form of CQS score described above. However, it turns out that, most erroneous variants remaining after UMI-based consensus assembly are characterized by high CQS quality (**S2B Fig**).

These errors could not arise at the stage of sequencing, as demonstrated on the following extreme example. Consider data with an average Phred quality of 20 (~1 error per 100 reads at a given position) and 5 reads per UMI threshold. The resulting theoretical probability that an error will become a dominant variant and emerge in the UMI consensus is 10^−5^, which is far lower than the observed erroneous variant size distribution (**S2C Fig**). Thus it is clear that errors remaining after UMI-assembling errors are not sequencing errors, and the probability of erroneous variant call is not correlated with major nucleotide frequency.

It is important to note that running conventional software tools such as VarScan and MuTect for assembled consensuses is unfeasible: telling real mutations from PCR and sequencing error noise is a crucial part of variant caller which relies on sequencing quality. However, the quality scores of assembled consensuses should not be confused with sequencing quality scores having different meaning and distribution. Therefore these scores will not work properly with conventional variant caller’s error model. As for the raw data, background sequencing error rate surpasses the 0.1% frequency threshold and complicate calling mutations of 0.1–1% frequency.

MAGERI implements a Beta-Binomial model for handling PCR errors and assigning variant quality scores. The model is fitted to error rates observed for six substitution types (A>C/T>G, A>G/T>C, A>T/T>A, C>A/G>T, C>G/G>C, C>T/G>A) in a pooled dataset that contains data from UMI-tagged sequencing experiments performed for a known template sequence and 9 different polymerases. A complete description of error model can be found here:

[https://github.com/mikessh/mageri-paper/blob/master/error_model/basic_error_model.pdf].

the datasets available in SRA under the accession PRJNA352143 are to be published elsewhere. Briefly, error frequencies for each substitution type are fitted with a Beta distribution (**S3 Fig**),

$$\epsilon_{xy}\sim Beta(\alpha_{xy},\beta_{xy});x,y\in \{A,T,G,C\}$$

observed error count *n*_*xyi*_ under the coverage *N*_*i*_ at a given position *i* is then modelled with a Beta-Binomial distribution,

$$n_{xyi}\sim BetaBinom(N_{i},\alpha_{xy},\beta_{xy})$$

which shows a good fit for errors observed in control healthy donor PBMC DNA (**S3B Fig**). MAGERI Q scores are computed as transformed Beta-Binomial P-values

$$Q=-10\;\log_{10}\;P_{BetaBinom}\;(n_{xyi},N_{i},\alpha_{xy},\beta_{xy})$$

To avoid floating point arithmetic issues, we have capped Q score calculation by setting a maximum Q score of 100 (P = 10^−10^).

The model assumes that PCR errors are introduced at the UMI tagging step. In case UMI attachment does not involve a PCR reaction (e.g. using ligation), the model can be adjusted to account for errors coming from the following PCR amplification. The probability of PCR error in this case should be adjusted by multiplying by the probability of a 1st cycle PCR error propagating to become a dominant variant within the consensus sequence due to PCR inefficiency and stochastics (**S4 Fig**) as follows:

$$\beta_{xy}^{-1}:=\beta_{xy}^{-1}∙\lambda^{2}(1-\lambda )$$

We should also note that it is possible to infer error rate by inspecting minor errors, i.e. errors found in reads that did not make it to the final consensus sequence after MIG assembly. This method relies on errors produced at early PCR cycles and requires good sequencing quality, high number of molecules and relatively high MIG size (UMI coverage) to perform robustly (which is not always reachable, e.g. in cases using MiSeq instrument with relatively low number of sequencing reads). The description and benchmark of the minor-based error model can be found at [https://github.com/mikessh/mageri-paper/blob/master/error_model/minor_based_error_model.pdf].

### Duplex sequencing data analysis

We have downloaded raw datasets from SRA (run accession SRR1799908) and preprocessed the data using “NNNNNNNNNNNNtgact” / “agtcaNNNNNNNNNNNN” primer patterns for demultiplexing and used all ABL1 exon sequences with 100 bp overhangs for alignment. The analysis is using default MAGERI parameters, not accounting for information from both consensus sequences, with the only adjustment that involves the error probability which was multiplied by the 1st cycle PCR propagation factor described above (PCR efficiency was set to 1.8).

### HIV amplicon sequencing data analysis

We have downloaded HIV-1 protease gene amplicon sequencing data reported in Ref. [37] from SRA (SRP052322). Datasets were pre-processed using “NNNNNNNNNcagtttaacttttgggccatccattcc” / “ctatcggctcctgnnnn” primer patterns and protease gene reference for HXB2 HIV-1 genome assembly obtained using Sequence Locator tool (http://www.hiv.lanl.gov/content/sequence/LOCATE/locate.html). Note that these libraries were prepared using RT-PCR and sequenced using Illumina MiSeq instrument in contrast to previously mentioned datasets. Default MAGERI parameters were used.

### IonTorrent sequencing data analysis

IonTorrent data was obtained from [38] and processed using default MAGERI parameters except for Torrent/454 settings preset for the consensus assembler: reads that have three or more consequent mismatches compared to the consensus sequence (indicating the presence of indels) were discarded and re-aligned using Smith-Waterman local alignment. UMI sequences from the header of available FASTQ file were used. The only dataset available for the study [http://datadryad.org/resource/doi:10.5061/dryad.n6068] contains UMI-tagged sequencing results for cloned *FGFR3* exon 7 template sequence. The reported control variant (R248C) is just 1 base away from the first base of the template and was not detected in reads.

## Results

### MAGERI benchmark using reference standard library

To test the accuracy of MAGERI pipeline we have selected a mutation reference standard with known somatic variant frequencies (Horizon Dx, Cambridge, UK) that was previously used for similar tasks [39,40] as a gold-standard dataset that can be used to assess the accuracy of UMI-tagged data processing and ultra-rare variant calling software. Reference standard was either used as-is or mixed with healthy donor PBMC DNA in 1:9 ratio to obtain a spectrum of known variants with different frequencies (listed in **S2 Table**) that were grouped into three tiers (0.1%, 1% and 5+%, listed in **S5 Table**), while healthy donor DNA alone served as a negative control.

UMI-tagged target amplicon libraries were generated using multiplex PCR amplification of genomic regions (**S1 Fig, S1 Table**) carrying mutations known to be present in the mutation reference standard. Resulting UMI-tagged libraries were then subject to deep sequencing on Illumina HiSeq2500 platform (Raw sequencing data: PRJNA297719) yielding on average 16,073,484+/-7,149,885 reads per sample. Primers and UMI base positions were identifiable for 87+/-4% of reads; UMI coverage distribution showed a clear peak (**S2A Fig**) sufficient for optimal error correction. The fraction of reads that belong to high-coverage UMIs and were successfully assembled was 99.9+/-0.3%, resulting in 33,911+/-14,203 consensus sequences, 98+/-4% of which were aligned to reference. A comprehensive MAGERI processing summary is provided in **S3 Table**.

**Fig 2:**
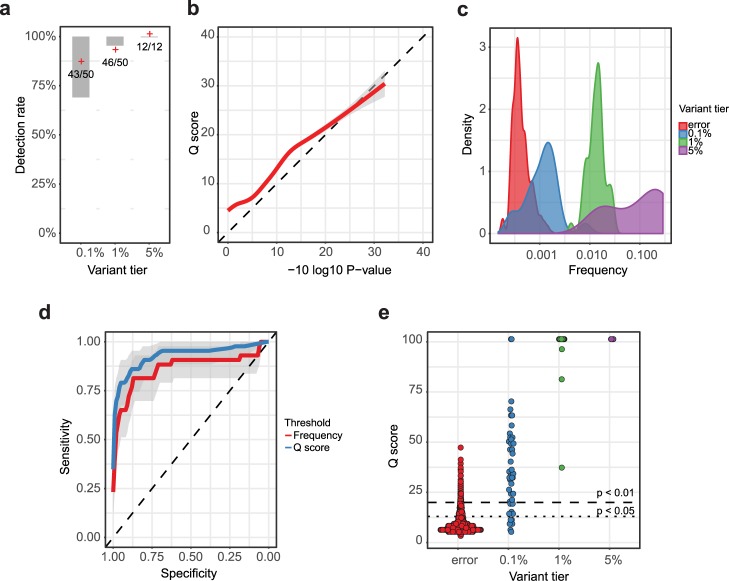
MAGERI software benchmark using Tru-Q 7 reference standard and control donor DNA. **a** Number of detected variant for each variant frequency tier across two independent experiments with the reference standard. Shaded areas show the 95% confidence intervals for expected fraction of recovered variants, i.e. binomial proportion confidence intervals built using known variant frequency and template coverage. **b** Frequency distribution of known Tru-Q 7 variants coming from each frequency tier and errors in the control donor DNA. **c** MAGERI Q score and the empirical P-values of erroneous variants detected in control donor DNA. **d** Comparison of Q score distribution of erroneous variants and variants of each frequency tier. Dotted and dashed lines show P < 0.05 and P < 0.01 thresholds respectively. **e** Receiver operation characteristic (ROC) curve comparing the sensitivity and specificity of MAGERI Q scores (blue line) and frequency-based thresholding (red line) in the task of classification of errors and 0.1% tier variants.

The number of variants that were identified by MAGERI prior to any variant quality filtering was in a good agreement with the one expected from low-frequency template sampling stochastics arising due to limited coverage (**Fig 2A**). Overall, variant frequencies obtained by MAGERI were in good agreement with known variant frequencies provided by the manufacturer (**Fig 2B**, Spearman R = 0.83, n = 101 accounting for all variant tiers, independent replicas and ignoring variants that were not detected). MAGERI variant quality scores (Q scores) for errors observed in healthy donor DNA were also in a good agreement with empirical P-values computed based on error frequencies (**Fig 2C**, Pearson R = 0.83, n = 2468). MAGERI Q scores for errors observed in control dataset and known variants from reference standard are shown in **Fig 2D**. These Q scores display a high area under curve (AUC) value when used as a threshold to classify errors and 0.1% tier variants (AUC = 93%, CI95: 87–98%, 2468 control and 43 cases), which is significantly better than the one obtained when using observed variant frequency as a threshold (AUC = 86%, CI95: 78–94%, **Fig 2E**).

### MAGERI performance in circulating tumor DNA detection

**Fig 3:**
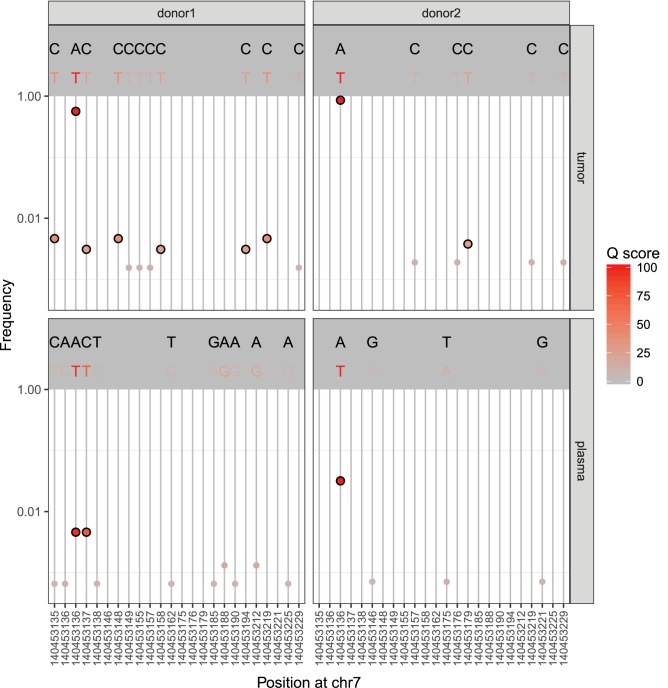
Detection of BRAF gene variants in tumor and plasma samples from two cancer patients. Each point represents a variant and is colored according to MAGERI Q score, upper panel of each plot shows reference (top) and variant (bottom) bases. Variants passing Q 20 threshold (P < 0.01) are shown with bold circles. Chromosome position is given in hg19 assembly coordinates.

To demonstrate applicability of MAGERI software to the analysis of patient samples we decided to tackle the problem of detecting circulating tumor DNA (ctDNA) [16] in peripheral blood of cancer patients. We have sequenced tumor and blood plasma DNA samples from two patients with locally advanced malignant skin melanoma using the UMI-based library preparation protocol described in **Materials and Methods** and ran MAGERI pipeline with default settings. We focused on variant calling results for the exon 15 of *BRAF* gene since both tumors were known to harbor the *BRAF* c.1798G>A (*BRAF* V600E[41]) mutation. The c.1798G>A mutation was detected in both patients’ plasma DNA at a frequency of 0.4% and 3.3% (**Fig 3**). Notably, the first patient’s plasma appear to contain the c.1799T>A mutation at 0.4% frequency, that is detected jointly (i.e. in the same MIGs) with c.1798G>A and together comprise the *BRAF* V600K variant[41] (**Fig 3**). The c.1799T>A variant is also present in the corresponding tumor sample, albeit at a far smaller frequency than c.1798G>A. The probability of jointly detecting this mutation pair simply by chance is P < 10^−18^ (Hypergeometric test), thus the first patient demonstrates an interesting case of a rare subpopulation of tumor cells that is dominant in ctDNA.

### MAGERI analysis of UMI-tagged libraries prepared using distinct methodologies

**Fig 4:**
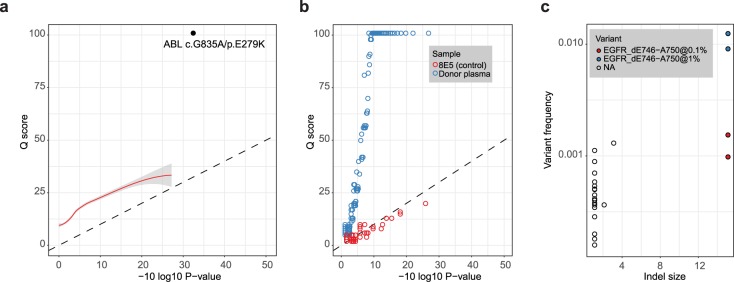
MAGERI performance on different types of UMI-tagged data. **a.** Analysis of single-strand consensuses from duplex sequencing data. Q scores of detected variants are plotted against empirical P-values, a smoothed fitting is shown with red line, ABL variant known to be present in the sample at ~1% frequency is shown with black dot. **b**. Analysis of UMI-tagged HIV cDNA sequencing data. MAGERI Q scores are plotted against empirical P-values for a control unmutated HIV cDNA from 8E5 cell line (red) and HIV+ donor plasma sample (blue). **c**. Indel variants detected in Tru-Q 7 reference standard and PBMC DNA of a healthy donor. Indel frequency is plotted against its size (number of added/deleted nucleotides). The figure shows known EGFR deletion (ΔE746 − A750) in two independent experiments with a known frequency of 1% (original Tru-Q 7 reference standard) and 0.1% (Tru-Q 7 reference standard diluted in 1:9 ratio with healthy donor DNA), erroneous variants present in healthy donor DNA are shown with empty circles.

For the sake of an independent validation we have applied our pipeline to a dataset from a recently published study[42,43] on duplex (double-stranded consensus) sequencing, an approach shown to be the most sensitive and specific among the currently existing UMI-based methods. This method relies on matching variants coming from both DNA strands tagged with the same UMI to boost variant calling accuracy and eliminate errors. Interestingly, even when operating with single-strand consensuses only (see **Materials and Methods**, *Duplex sequencing data analysis* section for details), we were able to reliably call a specific ABL1 mutation used by Schmitt *et al*. as a control at 0.8% frequency, while MAGERI Q scores were in a good agreement with empirical P-values for remaining erroneous variants (**Fig 4A**). As the duplex sequencing dataset uses ligation for UMI attachment, Q-scores were adjusted to account for the probability of 1st cycle PCR error propagation to become a dominant variant within the consensus (see **Materials and Methods**, *Variant calling* section). It is necessary to note that the setup that includes just a single test variant with a frequency that by far exceeds that of the most abundant errors is inadequate for performing a comprehensive rare mutation calling benchmark. Nevertheless, MAGERI was able to reliably quantify the distribution of error frequencies in the described case. Using MAGERI and single-strand consensus sequencing can be beneficial, as duplex consensus pairing results in a dramatic decrease of coverage: we observed a median of ~7000 consensuses per position for single-strand molecules and only 1000 consensuses for double-stranded molecules, which is far more than the expected 2x loss.

To demonstrate the versatility of our software pipeline, we have additionally tested it using a dataset from a completely different domain, HIV amplicon sequencing recently published by Zhou et al.[37] (see **Materials and Methods**). MAGERI was able to successfully process data coming from a cDNA-based library sequenced with error-prone long reads with no parameter modifications. Q scores computed by MAGERI for erroneous variants detected in HIV cDNA from 8E5 cell line which serves as a control in this experiment were in good agreement with empirical P-values computed from variant frequencies (**Fig 4B**, red dots). On the other hand, HIV cDNA from patient sample that should contain a wealth of mutations displays a drastically different picture with many high-quality variants (**Fig 4B**, blue dots).

### Indel detection and indel-prone sequencing data

Erroneous insertions and deletions (indels) at homopolymers are common in high-throughput sequencing performed using Roche 454 and Ion Torrent instruments [44,45], and a detectable fraction of such errors is generated by Illumina instruments [46]. While quality filtering of indel calls is out of scope of current paper, we suggest that UMI-tagged sequencing will greatly decrease the burden of indel errors and have implement the ability to output indel variants in MAGERI pipeline. The results of indel calling in Tru Q 7 reference standard dataset and healthy donor DNA show that the assembled consensus sequences still contain a fraction of short indel errors, yet the known deletion in *EGFR* gene can be reliably detected at both 1% and 0.1% frequency (**Fig 4C**).

We have additionally tested the ability to assemble the indel-prone Ion Torrent data published in Ref. [38] (see **Materials and Methods**). Presence of indels in sequencing reads had little effect on the overall assembly efficiency and more than 99.9% of reads successfully assembled into consensuses. Erroneous indels observed in the sequencing data from a cloned *FGFR3* exon 7 template can be efficiently filtered by increasing the MIG size threshold: 3 deletions are observed at 5 reads per UMI threshold, 2 deletions are observed at 10–15 reads threshold, and no indels are observed at 20+ threshold. It should be noted, however, that as MAGERI does not implement any indel quality assessment algorithm, indel calls should be manually checked for alignment artefacts and strand bias using MAGERI output in SAM format.

## Discussion

The results obtained with MAGERI can be used in a wide range of downstream analyses, such as variant effect annotation[47], comparison with variant databases such as COSMIC and dbSNP that can greatly improve reliability of variant calling, or somatic mutation phasing[48]. The latter, as we believe, will benefit much from the improvement in variant quantification gained from template counting capabilities of UMI tags.

It is important to stress the fact that MAGERI implements a control-free rare variant caller. In this sense it differs from the majority of somatic variant calling tools that aim at distinguishing somatic variants of moderate frequency in homogenous tumor samples from germline mutations and thus require a matched control sample[11]. In case of UMI-assembled data that has low error rates the main focus is placed on calling rare variants which are unambiguously somatic. High-frequency somatic variants are straightforward to obtain by subtracting variants found in control sample.

MAGERI fills an important gap in genome re-sequencing analysis software family and allows easy and efficient processing of high-throughput sequencing data generated using UMI-based protocols. This software represents a solution for a wide range of applications requiring high-accuracy rare variant detection such as tumor genomic heterogeneity studies, translational studies involving ctDNA detection and discovery of rare resistant variants by viral amplicon sequencing.

## Supporting information

S1 Table Genes, regions, and primer sequences. (XLS)

S2 Table Known Tru-Q 7 1% Tier standard variants used for MAGERI benchmark. The table contains coordinates in hg19 assembly, variant type and name, and variant frequency as reported by the vendor. Note that all variants are assayed in two independent experiments and two dilutions (1X and 0.1X).
(PDF)

S3 Table Processing statistics for Tru-Q 7 reference standard and healthy donor DNA. The table contains sample name, experiment type (standard for Tru-Q 7 and blank for control DNA), primer set (m1 − 4) used for amplicon sequencing, the ID of independent experiment (replica). The statistics include: total number of reads, fraction of reads in which the UMI and both forward and reverse primers were found unambiguously, number of unique UMIs and number of MIGs that had enough coverage and were successfully assembled into consensus sequences, fraction of reads in assembled UMIs and the total number of aligned consensuses.
(PDF)

S4 Table Benchmark of K-mer based reference selection algorithm. (PDF)

S5 Table Total number of variants in each frequency tier. Total number of variants in each frequency tier coming from two independent experiments and two dilutions (1X and 0.1X) of Tru-Q 7 reference standard.
(PDF)

S1 Fig ctDNA library preparation outline. UMI tagging is ensured by five cycles of linear PCR. Tagging primer is digested by ExoI treatment. Following steps comprise a combination of nested (R3, R4-Int) and step-out (F2, F4-Ext primers) amplification. Illumina adapters for TruSeq sequencing and flow-cell attachment oligonucleotides are included during amplification. During the last step, sample index is inserted for the aims of demultiplexing of different libraries.
(PDF)

S2 Fig UMI extraction and variants in consensus sequences for Tru-Q 7 reference standard and healthy donor DNA. **a.** MIG size distribution, total number of reads in MIGs of a specific size. Each sample is shown with color, two independent experimental replicas are shown as solid and dashed lines. **b.** Histogram of consensus quality scores (share of major base in consensus scaled to 0–40 range) for erroneous variants found in healthy donor DNA. **c.** Histogram of MIG counts of errors observed in healthy donor DNA and error counts expected from sequencing errors under 5 read MIG size threshold and a sequencing quality Phred score of 20.
(PDF)

S3 Fig Fitting a model of PCR error frequencies. **a.** Fitting Beta distribution to error frequencies observed in UMI-tagged sequencing experiment of a template with a known sequence. Grey area shows the density of observed error frequencies, red line shows the fitting. **b.** Error counts observed in UMI- tagged sequencing of healthy donor DNA that should (black line and points) and expected from the fitted Beta-Binomial model (red line).
(PDF)

S4 Fig Estimating the probability of first-cycle PCR error becoming a dominant variant among PCR products of DNA molecule tagged with an UMI tag. Here *epsilon* is the error probability and *lambda* is the PCR efficiency minus one.
(PDF)
