# Supplementary material for: MAGERI: Computational pipeline for molecular-barcoded targeted resequencing
Source: PLoS Comput Biol. 2017 May 5;13(5):e1005480. doi: 10.1371/journal.pcbi.1005480 (PMC5419444; doi:10.1371/journal.pcbi.1005480)
Supplement: S2 Table — The table contains coordinates in hg19 assembly, variant type and name, and variant frequency as reported by the vendor. Note that all variants are assayed in two independent experiments and two dilutions (1X and 0.1X). (PDF) [file pcbi.1005480.s002.pdf]

**Chr**	**Position**	**From**	**To**	**Variant**	**Known frequency**	**Reference**
chr7	140453136	A	T	BRAF_V600E,BRAF_V600K	0.08	BRAF_E15
chr7	140453136	A	C	BRAF_V600G,BRAF_V600R	0.01	BRAF_E15
chr7	140453137	C	T	BRAF_V600M,BRAF_V600K,BRAF_V600R	0.01	BRAF_E15
chr7	55241707	G	A	EGFR_G719S	0.167	EGFR_E18
chr7	55259515	T	G	EGFR_L858R	0.01	EGFR_E21
chr7	55259524	T	A	EGFR_L861Q	0.01	EGFR_E21
chr7	55249071	C	T	EGFR_T790M	0.01	EGFR_E20
chr19	3118942	A	T	GNA11_Q209L	0.013	GNA11_E5
chr2	209113112	C	T	IDH1_R132C	0.013	IDH1_E4
chr2	209113113	G	A	IDH1_R132H	0.013	IDH1_E4
chr12	25378562	C	T	KRAS_A146T	0.013	KRAS_E4
chr12	25398284	C	G	KRAS_G12A	0.013	KRAS_E2
chr12	25398285	C	A	KRAS_G12C	0.013	KRAS_E2
chr12	25398284	C	T	KRAS_G12D	0.013	KRAS_E2
chr12	25398285	C	G	KRAS_G12R	0.013	KRAS_E2
chr12	25398285	C	T	KRAS_G12S	0.013	KRAS_E2
chr12	25398284	C	A	KRAS_G12V	0.013	KRAS_E2
chr12	25398281	C	T	KRAS_G13D	0.25	KRAS_E2
chr12	25380275	T	G	KRAS_Q61H	0.013	KRAS_E3
chr12	25380276	T	A	KRAS_Q61L	0.013	KRAS_E3
chr1	115256528	T	A	NRAS_Q61H	0.013	NRAS_E3
chr1	115256530	G	T	NRAS_Q61K	0.013	NRAS_E3
chr1	115256529	T	A	NRAS_Q61L	0.013	NRAS_E3
chr1	115256529	T	C	NRAS_Q61R	0.013	NRAS_E3
chr3	178936082	G	A	PIK3CA_E542K	0.013	PIK3CA_E9
chr3	178936091	G	A	PIK3CA_E545K	0.013	PIK3CA_E9
chr3	178952085	A	G	PIK3CA_H1047R	0.3	PIK3CA_E20
chr7	55242465	GGGAATTAAGAGAAGC	G	EGFR_dE746-A750	0.01	EGFR_E19
