# Supplementary material for: MAGERI: Computational pipeline for molecular-barcoded targeted resequencing
Source: PLoS Comput Biol. 2017 May 5;13(5):e1005480. doi: 10.1371/journal.pcbi.1005480 (PMC5419444; doi:10.1371/journal.pcbi.1005480)
Supplement: S3 Table — The table contains sample name, experiment type (standard for Tru-Q 7 and blank for control DNA), primer set (m1 − 4) used for amplicon sequencing, the ID of independent experiment (replica). The statistics include: total number of reads, fraction of reads in which the UMI and both forward and reverse primers were found unambiguously, number of unique UMIs and number of MIGs that had enough coverage and were successfully assembled into consensus sequences, fraction of reads in assembled UMIs and the total number of aligned consensuses. (PDF) [file pcbi.1005480.s003.pdf]

Dataset	Primer set	Rep-lica	Variant ratio	Reads	UMI found, %	Unique UMIs	Consen-suses	Assembled reads,%	Aligned consensus es	Mapping rate
Control DNA	1	1	NA	13062691	84%	45653	45599	100%	45146	99%
Control DNA	2	1	NA	15530074	90%	50454	50382	100%	50372	100%
Control DNA	3	1	NA	15161566	89%	46911	46857	100%	46819	100%
Control DNA	4	1	NA	32156954	86%	28304	28271	100%	27603	98%
Control DNA	1	2	NA	13726772	84%	46178	46108	100%	45617	99%
Control DNA	2	2	NA	15958155	90%	47922	47857	100%	47833	100%
Control DNA	3	2	NA	13952806	89%	41151	41102	100%	41062	100%
Control DNA	4	2	NA	32327434	87%	27648	27619	100%	27259	99%
Tru-Q 7	1	1	0.1	17460396	86%	53269	53178	99%	52777	99%
Tru-Q 7	2	1	0.1	17422223	91%	38495	38437	100%	38433	100%
Tru-Q 7	3	1	0.1	14055586	89%	48040	48007	100%	48005	100%
Tru-Q 7	4	1	0.1	26551241	83%	34643	34615	100%	32434	94%
Tru-Q 7	1	1	1	10258304	85%	20871	20817	100%	20354	98%
Tru-Q 7	2	1	1	10794271	91%	18937	18880	100%	18846	100%
Tru-Q 7	3	1	1	5417463	89%	17475	17463	100%	17452	100%
Tru-Q 7	4	1	1	18957455	75%	14578	14567	100%	12608	86%
Tru-Q 7	1	2	0.1	16075588	86%	50960	50863	99%	50431	99%
Tru-Q 7	2	2	0.1	20053163	90%	39658	39594	100%	39583	100%
Tru-Q 7	3	2	0.1	11839770	89%	50911	50873	100%	50871	100%
Tru-Q 7	4	2	0.1	25367956	85%	29377	29354	100%	27637	94%
Tru-Q 7	1	2	1	8618544	85%	19998	19942	100%	19457	97%
Tru-Q 7	2	2	1	10570707	90%	17148	17096	100%	17076	100%
Tru-Q 7	3	2	1	4880473	90%	13483	13468	100%	13464	100%
Tru-Q 7	4	2	1	15564034	78%	12922	12907	100%	11206	87%
