# Supplementary material for: MAGERI: Computational pipeline for molecular-barcoded targeted resequencing
Source: PLoS Comput Biol. 2017 May 5;13(5):e1005480. doi: 10.1371/journal.pcbi.1005480 (PMC5419444; doi:10.1371/journal.pcbi.1005480)
Supplement: S4 Table — (PDF) [file pcbi.1005480.s004.pdf]

Benchmark type	Edit distance between reference sequences (1/bp)	Error rate in reads (1/bp)	Correct hits (%)
Highly-homologous references	0.01	0.01	95
Homologous references	0.1	0.01	100
Non-homologous references, high error rate	1.0	0.1	99
