# Supplementary material for: MAGERI: Computational pipeline for molecular-barcoded targeted resequencing
Source: PLoS Comput Biol. 2017 May 5;13(5):e1005480. doi: 10.1371/journal.pcbi.1005480 (PMC5419444; doi:10.1371/journal.pcbi.1005480)
Supplement: S5 Table — Total number of variants in each frequency tier coming from two independent experiments and two dilutions (1X and 0.1X) of Tru-Q 7 reference standard. (PDF) [file pcbi.1005480.s005.pdf]

**Variant tier**	**Variant frequency**	**Number of variants**
0.1%	<=0.9%	50
1%	>0.9%,<=2%	50
5+%	>2%	12
