# Supplementary figures and images for: MAGERI: Computational pipeline for molecular-barcoded targeted resequencing
Source: PLoS Comput Biol. 2017 May 5;13(5):e1005480. doi: 10.1371/journal.pcbi.1005480 (PMC5419444; doi:10.1371/journal.pcbi.1005480)

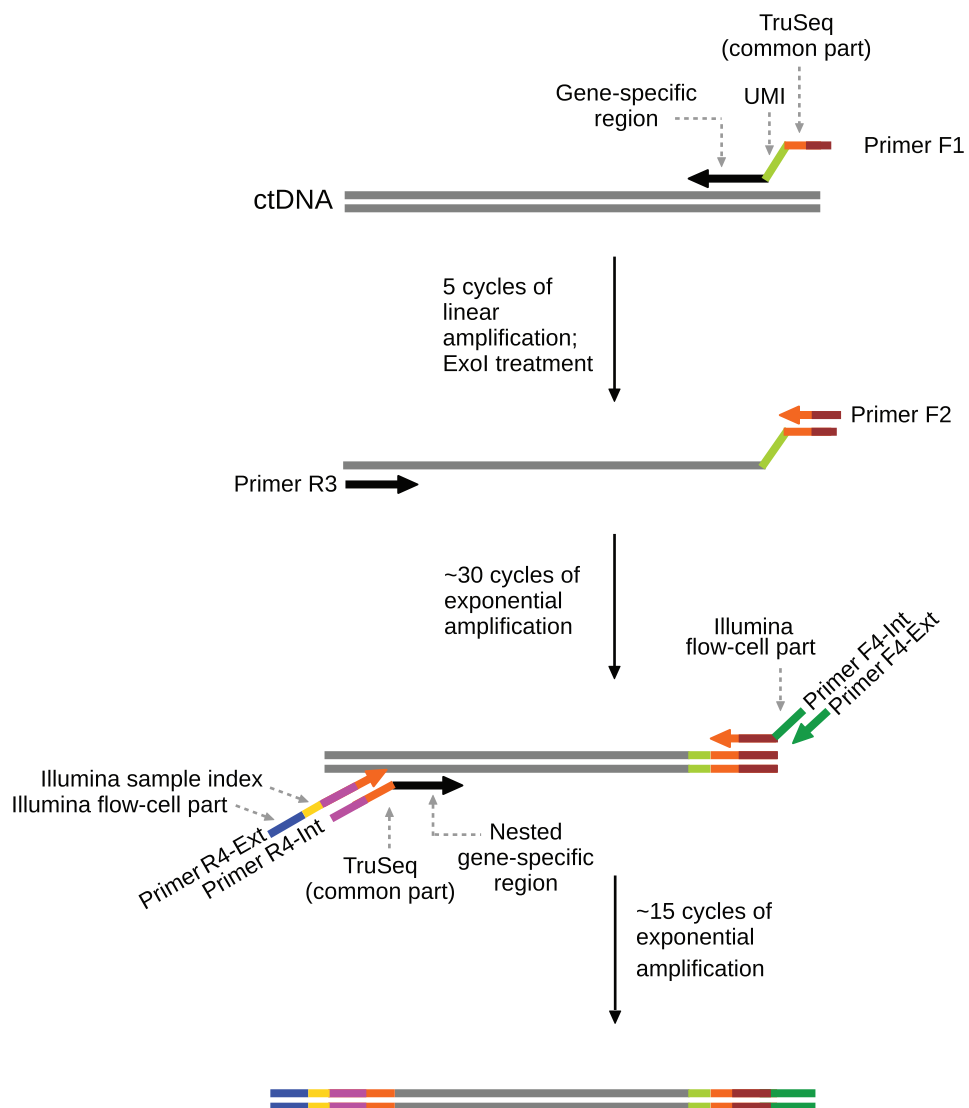

Supplement: S1 Fig — UMI tagging is ensured by five cycles of linear PCR. Tagging primer is digested by ExoI treatment. Following steps comprise a combination of nested (R3, R4-Int) and step-out (F2, F4-Ext primers) amplification. Illumina adapters for TruSeq sequencing and flow-cell attachment oligonucleotides are included during amplification. During the last step, sample index is inserted for the aims of demultiplexing of different libraries. (PDF) [file pcbi.1005480.s006.pdf]

**a**

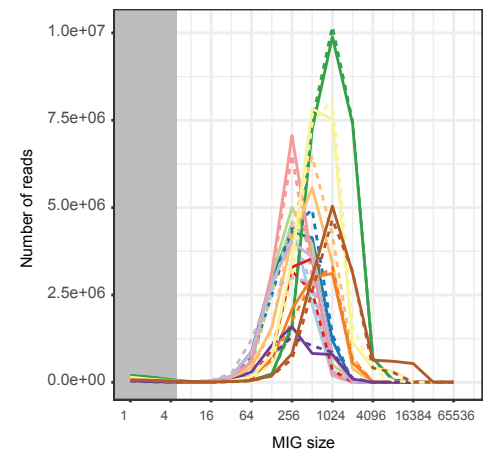

**b**

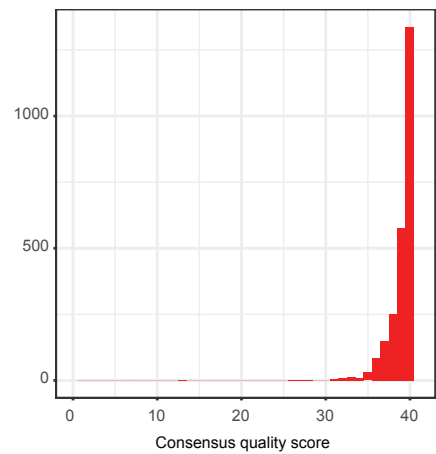

**c**

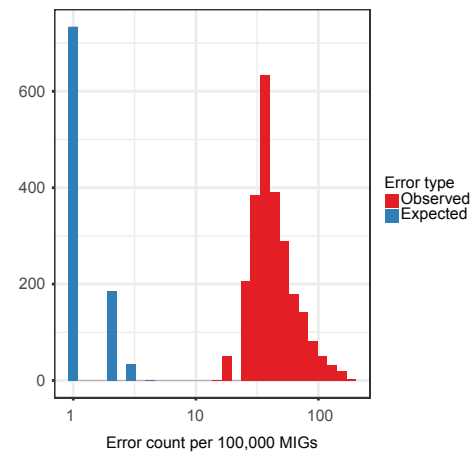

Supplement: S2 Fig — a. MIG size distribution, total number of reads in MIGs of a specific size. Each sample is shown with color, two independent experimental replicas are shown as solid and dashed lines. b. Histogram of consensus quality scores (share of major base in consensus scaled to 0–40 range) for erroneous variants found in healthy donor DNA. c. Histogram of MIG counts of errors observed in healthy donor DNA and error counts expected from sequencing errors under 5 read MIG size threshold and a sequencing quality Phred score of 20. (PDF) [file pcbi.1005480.s007.pdf]

**a**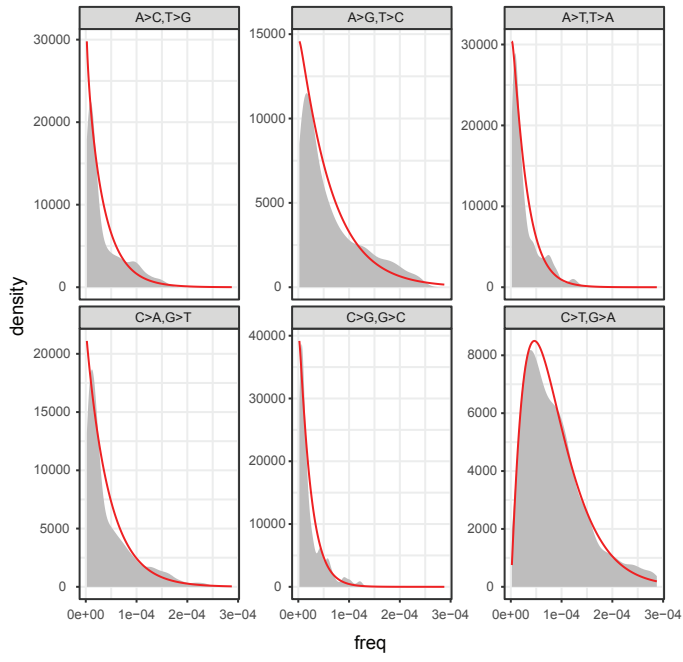**b**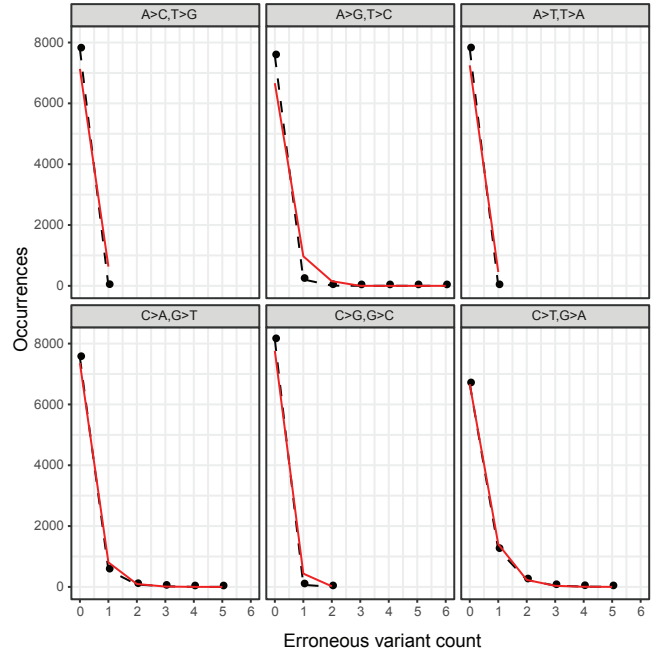

Supplement: S3 Fig — a. Fitting Beta distribution to error frequencies observed in UMI-tagged sequencing experiment of a template with a known sequence. Grey area shows the density of observed error frequencies, red line shows the fitting. b. Error counts observed in UMI- tagged sequencing of healthy donor DNA that should (black line and points) and expected from the fitted Beta-Binomial model (red line). (PDF) [file pcbi.1005480.s008.pdf]

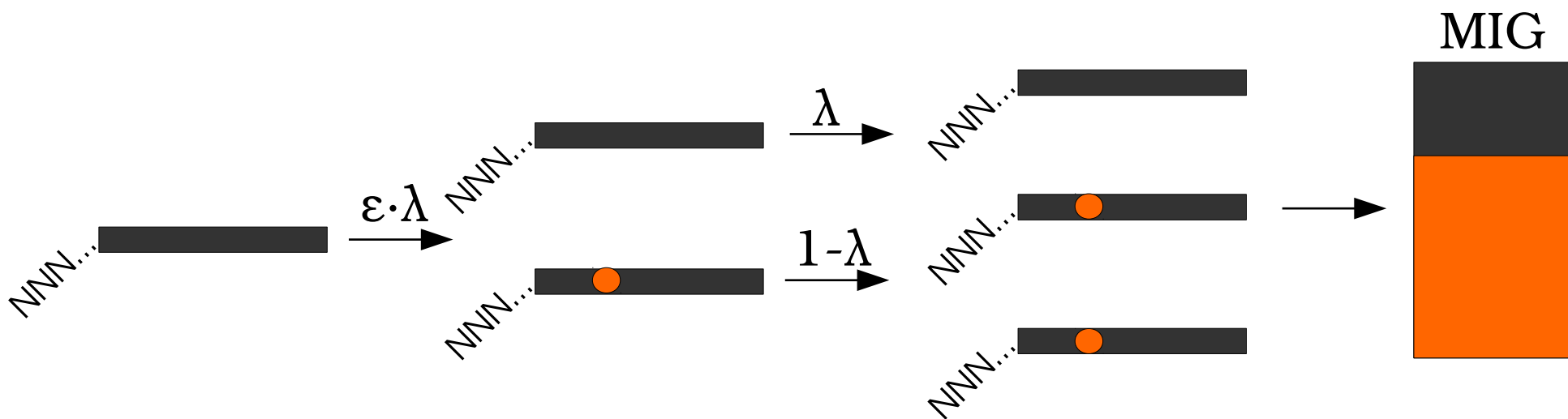

Supplement: S4 Fig — Here epsilon is the error probability and lambda is the PCR efficiency minus one. (PDF) [file pcbi.1005480.s009.pdf]
